# Supplementary material for: Apolipoprotein CIII Reduction Protects White Adipose Tissues against Obesity-Induced Inflammation and Insulin Resistance in Mice
Source: Int J Mol Sci. 2021 Dec 22;23(1):62. doi: 10.3390/ijms23010062 (PMC8744831; doi:10.3390/ijms23010062)
Supplement: Supplementary file 1 [file ijms-23-00062-s001.zip › ijms-1508617-supplementary.pdf]

Supplementary Material

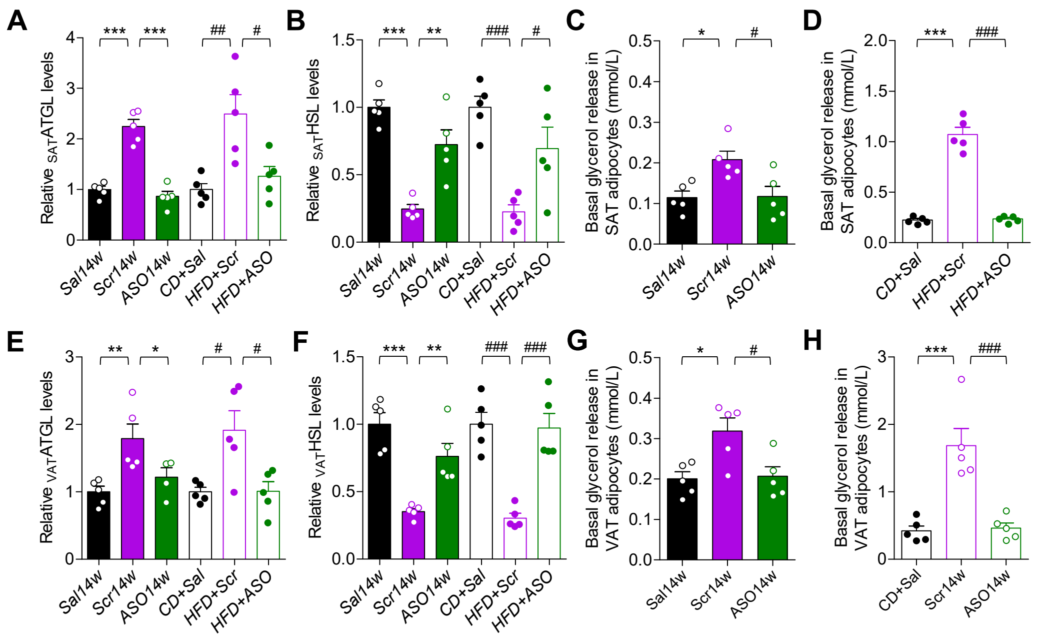

Figure S1. Gene expression of intracellular lipases in WATs and basal glycerol release in isolated adipocytes from SAT and VAT. (**A,B**) Gene expression in SAT (**A**) ATGL and (**B**) HSL by qRT-PCR. (**C,D**) Basal glycerol release in adipocytes from SAT from animals in the (**C**) prevention and (**D**) reversibility studies. (**E,F**) Gene expression in VAT (**E**) ATGL and (**F**) HSL by qRT-PCR. (**G,H**) Basal glycerol release in adipocytes from VAT from animals in the (**G**) prevention and (**H**) the reversibility studies. Data are expressed as mean ± SEM of n = 4-5 individual mice per experimental group and controls. * $p < 0.05$; ** $p < 0.01$; *** $p < 0.001$ for differences in the prevention study. # p < 0.05; ## p < 0.001; ### p < 0.001 for differences in the reversibility study.
